# Risk Factors for Severe–Critical COVID-19 in Pregnant Women

**DOI:** 10.3390/jcm12185812

**Published:** 2023-09-07

**Authors:** María Guadalupe Berumen-Lechuga, Alfredo Leaños-Miranda, Carlos José Molina-Pérez, Luis Rey García-Cortes, Silvia Palomo-Piñón

**Affiliations:** 1 Medical Research Coordinator, Instituto Mexicano del Seguro Social, Órgano de Operación Administrativa Desconcentrada Regional Poniente, Josefa Ortiz de Domínguez S/N Colonia Centro, Toluca CP 50000, Mexico; 2 Medical Research Unit in Reproductive Medicine, Instituto Mexicano del Seguro Social, Unidad Médica de Alta Especialidad, Hospital de Ginecología y Obstetricia “Luis Castelazo Ayala”, Rio de la Magdalena 289, Tizapán San Ángel, Delegación Álvaro Obregón, Ciudad de México CP 01090, Mexico; alfredolm@yahoo.com; 3 Clinical Coordinator, Instituto Mexicano del Seguro Social, Hospital General de Zona No. 252, Camino Flor de María y Calzada Señor del Huerto 154, Colonia San Martín, Atlacomulco CP 54050, Mexico; carlosjosem@hotmail.com; 4 Medical Research Coordinator, Instituto Mexicano del Seguro Social, Órgano de Operación Administrativa Desconcentrada Regional Oriente, Calle 4 No. 25, Fraccionamiento Alce Blanco, Naucalpan CP 53370, Mexico; docluisrey@gmail.com; 5 Medical Research Unit on Nephrological Diseases (External Collaborator), Instituto Mexicano del Seguro Social, UMAE Centro Médico Nacional Siglo XXI, Avenida Cuauhtémoc 330, Colonia Doctores, Ciudad de México CP 06720, Mexico; silvia-palomo@hotmail.com

**Keywords:** COVID-19, SARS-CoV-2, coronavirus infections, pregnancy

## Abstract

Risk factors associated with severe–critical COVID-19 (coronavirus disease 2019) are based on findings in the general population. Pregnant women are at increased risk of severe–critical infection, and few reports are based on these women. A multicentric case–control study was conducted at the Mexican Institute of Social Security, State of Mexico, during the COVID-19 pandemic. We included pregnant women who were consecutively admitted to respiratory care units and were followed until 30 days after the resolution of pregnancy. A total of 758 pregnant women with a positive RT-PCR test for SARS-CoV-2 were enrolled from June 2020 to July 2021. We defined groups using the World Health Organization Severity Classification; cases were pregnant women with severe–critical COVID-19 (n = 123), and controls were subjects with non-severe COVID-19 (n = 635). Data was gathered from clinical files. A multivariate logistic regression analysis was used to adjust odds ratios and their 95% confidence intervals of factors associated with severe–critical COVID-19. Risk factors associated with severe–critical COVID-19 in pregnancy were non-vaccination (OR 10.18), blood type other than O (OR 6.29), maternal age > 35 years (OR 5.76), history of chronic hypertension (OR 5.12), gestational age at infection ≥ 31 weeks (OR 3.28), and multiparity (OR 2.80).

## 1. Introduction

The coronavirus disease 2019 (COVID-19) pandemic caused a global health crisis. It is well recognized that COVID-19 in pregnant women is more frequent due to physiological changes in the immune and cardiorespiratory systems that increase the susceptibility to this infection. Pregnant women have a high frequency in the progression of COVID-19 into a severe–critical stage compared to non-pregnant women; in addition, COVID-19 is generally associated with poor fetal and maternal outcomes [1,2,3,4,5]. Although different in pathology and transmission, the 2015–2016 Zika virus disease epidemic shares similarities with severe acute respiratory syndrome coronavirus 2 (SARS-CoV-2), such as limited diagnostic techniques, therapeutics, and prognostic uncertainties, and both are associated with a significant risk of adverse outcomes including intrauterine transmission [6,7].

In non-pregnant subjects, many risk factors have been identified in the progression of COVID-19 into a severe stage, such as chronic hypertension, diabetes, and obesity, among others [2,8,9,10,11]. This highlights that individuals with pre-existing comorbidities related to the imbalance in expressing angiotensin-converting enzyme 2 (ACE2) (which acts as an entry receptor for SARS-CoV-2) are at high risk of developing severe disease.

Although we have gained experience about the severity, course, and treatment of disease in patients infected with SARS-CoV-2, there are scarce studies on the risk factors for severe COVID-19 in pregnancy [12]. The studies have been consistent in that maternal age (greater than 35 years) and pre-existing comorbidities (mainly chronic hypertension, pregestational diabetes, and obesity) are associated with severe–critical COVID-19 [3,13,14]. In contrast, some risk factors, such as the trimester in which the disease appeared, parity or blood group, as well as other specific disorders of pregnancy, such as pre-eclampsia or gestational diabetes, have been inconsistent [3,13,15].

Therefore, the aim of this study was to identify the risk factors for severe–critical COVID-19 in Mexican pregnant women during the pandemic caused by this virus.

## 2. Materials and Methods

A case–control study was performed. Pregnant women with a positive reverse transcription polymerase chain reaction (RT-PCR) test were consecutively admitted to the medical respiratory units in general hospitals: 71, 72, 194, and 251 of the Mexican Institute of Social Security in the State of Mexico were included from 1 June 2020 to 31 July 2021. Disease severity was defined according to the World Health Organization Classification [13]. The cases group was formed by women who developed severe–critical COVID-19 defined by the following criteria: acute respiratory distress syndrome, sepsis, septic shock, thrombosis, or other conditions that would normally require life-sustaining therapies such as mechanical ventilation (invasive or non-invasive), vasopressor therapy, or oxygen saturation < 92% on room air; severe pneumonia; or signs of severe respiratory distress such as use of accessory muscles, inability to complete full sentences, and respiratory rate > 30 breaths per minute. The control group was formed by women with non-severe COVID-19, defined as the absence of any criteria for severe or critical COVID-19. Women whose diagnosis was not confirmed by RT-PCR, pregnant minors, those at risk of losing their autonomy, and those participants with incomplete data were eliminated. All women and newborns were followed until 30 days after the resolution of pregnancy.

Data were prospectively collected from medical charts. Differences between continuous variables were determined by Student’s *t*-test for unpaired data or Mann–Whitney U test for non-normally distributed variables. Differences between categorical variables were determined using the chi-squared test with Yates’ continuity correction or Fisher’s exact test for small samples. The association between the clinical and demographic variables reported as risk factors and the occurrence of severe–critical COVID-19 were determined. Odds ratios (ORs) and 95% confidence intervals (CIs) were calculated. Logistic regression analysis was used to adjust the ORs for maternal age > 35 years, maternal comorbidities (chronic hypertension, pregestational diabetes, and obesity), non-vaccination against COVID-19, maternal blood group, gestational age at infection, multiparity, C-reactive protein, D-dimer, and neutrophil-to-lymphocyte ratio, because these variables were significantly different among the groups studied by univariate analysis, as well as considering that this variable may distinctly affect the occurrence of severe–critical COVID-19. A 2-tailed *p* < 0.05 was considered statistically significant. SPSS version 27 statistical software was used for data analysis.

The study protocol was approved on 22 May 2020 by the National Committee of Scientific Research of the Mexican Institute of Social Security with SIRELCIS registry R-2020-785-067. Written informed consent was obtained from all participants.

## 3. Results

### 3.1. General Description of the Population Studied

A total of 1492 pregnant women were attended by the respiratory modules. Seven hundred and seventy-one (51.7%) had a positive RT-PCR test; among these patients, seven did not accept to participate, and six were eliminated due to incomplete medical charts. Therefore, 758 patients were included in the final analysis; 123 were diagnosed with severe–critical COVID-19, and 635 women had non-severe COVID-19.

#### 3.1.1. The Demographic and Clinical Characteristics Are Shown in Table 1

There was no difference between groups in terms of days to seek medical attention, smoking history during pregnancy, vaccine brand, pre-eclampsia, or gestational diabetes. In contrast, there was a significant difference in maternal age, multiparity, blood group (except AB), gestational age at infection, pulmonary involvement score on computed tomography, mechanical ventilation, vaccination, as well as in comorbidities (*p* ≤ 0.022). It is important to note that only 170 pregnant women were vaccinated because recruitment began before the vaccine was approved for use in pregnancy, all of whom were in their second or third trimester.

**Table 1:** Demographic and clinical characteristics of pregnant women with and without severe–critical COVID-19.

Variable	Cases	Controls	*p*-Value
	Severe–Critical COVID-19 [n = 123]	Non-Severe COVID-19 [n = 635]	
**Maternal age, years, median, [IQR]**	30 [26–34]	27 [23–31]	˂0.001 *
**Days to seek medical attention, median, [IQR]**	3 [2–6]	3 [2–5]	NS *
**Smoking history during pregnancy, n [%]**	6 [4.9]	26 [4.1]	NS †
**Multiparity, n [%]**	73 [59.3]	206 [32.4]	<0.001 †
**Mother’s blood group**			
O, n [%]	29 [23.6]	431 [67.9]	<0.001 †
A, n [%]	79 [64.2]	182 [28.7]	<0.001 †
B, n [%]	11 [8.9]	14 [2.2]	<0.001 †
AB, n [%]	4 [3.3]	8 [1.2]	NS ‡
**Maternal comorbidities**			
Chronic Hypertension, n [%]	12 [9.8]	13 [2.1]	<0.001 †
Pregestational Diabetes, n [%]	10 [8.1]	15 [2.4]	0.002 †
Obesity, n [%]	15 [12.2]	38 [6.0]	0.022 †
Pre-eclampsia, n [%]	26 [21.1]	173 [27.2]	NS †
Gestational Diabetes, n [%]	11 [9.1]	36 [5.65]	NS †
**Non-vaccination, n [%]**	117 [95.1]	471 [74.2]	<0.001 †
**Gestational age at infection, median, [IQR]**	33 [27–36]	23 [14–32]	<0.00 *
**Pulmonary involvement score in computed tomography**			
Noninvolvement, n [%]	0 [0]	427 [67.2]	<0.001 ‡
Mild, n [%]	0 [0]	80 [12.6]	<0.001 ‡
Moderate, n [%]	0 [0]	128 [20.2]	<0.001 ‡
Severe, n [%]	123 [100]	0 [0]	<0.001 ‡
**° Mechanical ventilation, n [%]**	32 [26.0]	4 [0.63]	<0.001 ‡
**°° Vaccination, n [%]**	9 [6.5]	161 [25.5]	<0.001 †
**Vaccine brand**			
Astra Zeneca, n [%]	3 [33.3]	27 [16.7]	NS ‡
Pfizer, n [%]	3 [33.3]	93 [57.9]	NS ‡
Sputnik, n [%]	2 [22.2]	25 [15.5]	NS ‡
Cansino, n [%]	0 [0]	5 [3.0]	NS ‡
Sinovac, n [%]	1 [11.1]	11 [6.7]	NS ‡

A *p*-value < 0.05 was considered statistically significant. * U Mann–Whitney test, † chi-squared test, ‡ Fisher’s exact test, ° the four pregnant women in the control group who needed mechanical ventilation had no relation with COVID-19. °° In vaccination, only 170 pregnant women received two doses because recruitment started before the vaccine authorization in pregnancy. No serious adverse events associated with vaccine administration occurred.

#### 3.1.2. The Main Clinical Signs and Symptoms and Laboratory Findings Are Shown in Table 2

No differences were found between groups except for frequency of anosmia and dysgeusia (*p* ≤ 0.17). As for laboratory findings, we found differences between groups in neutrophil-to-lymphocyte ratio, C-reactive protein, and D-dimer serum concentration (*p* ≤ 0.032).

**Table 2:** Main clinical manifestations and laboratory findings at admission in pregnant women with severe–critical versus non-severe COVID-19.

Variable	Cases	Controls	*p*-Value
	Severe–Critical COVID-19	Non-Severe COVID-19	
	[n = 123]	[n = 635]	
**Signs and symptoms**			
Headache, n [%]	89 [72.4]	463 [73.0]	NS †
Cough, n [%]	74 [60.2]	355 [55.6]	NS †
Odynophagia, n [%]	74 [60.2]	359 [56.4]	NS †
Fever, n [%]	71 [57.7]	320 [50.39]	NS †
Myalgias, n [%]	59 [48.0]	282 [44.5]	NS †
Anosmia, n [%]	12 [9.8]	124 [19.6]	0.003 †
Dysgeusia, n [%]	10 [8.1]	96 [15.1]	0.017 †
**Laboratory findings**			
Leukocytes, mean ± SD	9828 ± 3086	8479 ± 2357	NS ^§^
Neutrophil to lymphocyte ratio, median [IQR]	8.72 [6.8–12.4]	6.35 [4.1–9.2]	0.011 *
Platelets [×1000], median [IQR]	151 [111–233]	181 [151–243]	NS *
Glucose [mg/dL], median [IQR]	92 [81–124]	81 [67–96]	NS *
Creatinine [mg/dL], mean ± SD	0.96 ± 0.25	0.61 ± 0.16	NS ^§^
Lactic dehydrogenase [U/L], median [IQR]	296 [189–533]	209 [160–329]	NS *
D-dimer, ng/ml, median [IQR]	1956 [1181–3435]	1216 [518–2418]	0.032 *
C-reactive protein, mg/dL, mean ± SD	8.3 ± 6.8	2.1 ± 1.62	0.027 ^§^

Data are presented as count and frequency n [%], mean ± SD means standard deviation; IQR, interquartile range. A *p*-value *p* < 0.05 was considered statistically significant. † chi-squared test, § Student’s *t*-test, * U Mann–Whitney.

#### 3.1.3. Maternal and Perinatal Outcomes Are Reported in Table 3

There were no differences between groups in the frequency of miscarriages or cesarean sections. All women with severe–critical COVID-19 were admitted to intensive care units, and only six women in the group of controls. The need for admission to the intensive care units in the control group was not related to respiratory causes and was not associated with the severity of COVID-19 (four patients for early postpartum hemorrhage and two patients for met severity criteria for pre-eclampsia). All maternal deaths in the cases group were due to COVID-19 complications; in contrast, in the control group, they were due to postpartum hemorrhage after cesarean section. Interestingly, there were no maternal deaths in vaccinated pregnant women.

Regarding perinatal outcomes, we found differences between groups in gestational age at delivery and infant’s birth weight (*p* < 0.001). Premature delivery was more frequent in women with severe–critical COVID-19, as well as admission to the neonatal intensive care unit and perinatal deaths (*p* < 0.001). There were no differences between groups in the frequency of newborns below the 10th percentile of weight.

**Table 3:** Maternal and perinatal outcomes of pregnant women with and without severe–critical COVID-19.

Variable	Cases	Controls	*p*-Value
	Severe–Critical COVID-19	Non-Severe COVID-19	
	[n = 123]	[n = 635]	
**Maternal outcomes**			
Miscarriage, n [%]	3 [2.4]	9 [1.4]	NS ‡
Cesarean section, n [%]	105 [85.3]	555 [87.4]	NS †
Admission to Intensive Care Unit, n [%]	123 [100]	6 [0.95]	<0.001 †
Acute Kidney Injury, n [%]	19 [15.2]	6 [0.95]	<0.001 †
Maternal Deaths °, n [%]	9 [7.31]	2 [0.31]	<0.001 ‡
**Perinatal Outcomes**			
Gestational age at delivery, weeks, median [IQR]	36 [32–38]	38 [36–39]	<0.001 *
Infant’s birth weight, Kilograms, median [IQR]	2.40 [1.90–2.86]	2.88 [2.45–3.42]	<0.001 *
Premature delivery			
≤28 weeks, n [%]	9 [2.43]	1 [0.0]	<0.001 ‡
28 to 34 weeks, n [%]	25 [20.3]	17 [2.67]	<0.001 †
>34 to <37 weeks, n [%]	48 [39.0]	295 [46.9]	NS †
Admission to Neonatal Intensive Care Unit, n [%]	14 [11.3]	26 [4.0]	0.001 †
Birth weight < 10th percentile, n [%]	9 [7.31]	32 [5.0]	NS †
Perinatal death, n [%]	12 [9.7]	15 [2.4]	<0.001 †
30-day hospital discharge, n [%]	102 [82.9]	604 [95.1]	˂0.001 †

Data are reported as median and interquartile range [IQR]; count and percentage, n [%]. A *p*-value < 0.05 was considered statistically significant. † chi-squared test, ‡ Fisher’s exact test, * U Mann–Whitney’s test. ° Two maternal deaths occurred in the control group; they were not related to COVID-19, and they were due to obstetric hemorrhage.

#### 3.1.4. Factors Associated with Severe–Critical COVID-19 during Pregnancy

The associations between risk factors and the occurrence of severe–critical COVID-19 during pregnancy are exhibited in Table 4.

We calculated the crude odds ratios (ORs) and confidence intervals at 95% (CI 95%) for those variables reported to be consistently associated with severe COVID-19 (maternal age and maternal comorbidities) and those that showed significant differences in the univariate analysis. In multivariable logistic regression analysis, variables that remained as independent risk factors associated with the occurrence of severe–critical COVID-19 during pregnancy correspond to the non-vaccination against COVID-19, maternal blood group non-O, maternal age > 35 years, gestational age at infection, chronic hypertension, and multiparity (ORs ≥ 2.80).

We present a pre-vaccination analysis in Table 5, which shows that non-O blood group was the main risk factor for severe–critical COVID-19, followed by maternal age ≥ 35 years, chronic hypertension, multiparity, and gestational age at infection ≥ 31 weeks’ gestation (ORs ≥ 2.50).

## 4. Discussion

In this case–control study, we identified the risk factors for the occurrence of severe–critical COVID-19 in a large sample of Mexican pregnant women with a SARS-CoV-2 infection who were admitted to the respiratory units at the Mexican Institute of Social Security at the State of Mexico. We found that during the pre-vaccination period, the frequency of severe–critical COVID-19 was 19.5%, which is similar to the previously reported frequency of 20.2% [16,17]. Interestingly, the frequency of developing severe–critical COVID-19 after vaccination decreased significantly (at 5.2%). Moreover, in the group of vaccinated pregnant women, there were no cases of maternal death, which is consistent with the large number of studies that have reported that vaccination prevents the severe form of disease and deaths caused by COVID-19 [18]. We demonstrated that gestational age at infection ≥31 weeks was an independent risk factor for severe–critical COVID-19 (OR 3.28); in this vein, similar results were reported by Kalafat E. et al. (RR 3.64) [19]. In this regard, we agree with the notion that these results appear to be related to respiratory limitation due to changes caused by pregnancy rather than immunosuppressive aspects. As previously reported, we also found that maternal age > 35 years was another independent risk factor for severe–critical disease (OR 5.76) [13,19,20,21,22,23].

Although few studies have attempted to associate parity with infection and severity of COVID-19, there is a controversy on this issue [13,20,24]. Our result showed that multiparity was an independent risk factor (OR 2.80), although this was probably because multiparity usually occurs in older women with more frequent comorbidities.

In this regard, a history of comorbidities have been reported as risk factors for severity of COVID-19, including hypertension due to the imbalance between the two major pathways of the renin–angiotensin–aldosterone system (RAAS), with ACE2/Ang 1-7 downregulation and ACE2/Ang II upregulation. In pregestational diabetes, due to the expression of ACE2 increased in tissues of the lung, inflammation, insulin resistance, and altered immune modulation by an increase in pro-inflammatory cytokines, and in the case of obesity also due to ACE2 overexpression in adipocytes, a pro-inflammatory state, and a reduced lung capacity, may contribute to this increased risk... However, in our study population, after multivariate analysis, only a history of chronic hypertension remained as an independent risk factor (OR 5.12), which is like previous reports [13,16,20,21].

Gestational diabetes is suggested as a risk factor like DM2 associated with overexpression of ACE2 in lung tissue and insulin resistance; however, we found no differences between groups. In relation to pre-eclampsia, endothelial dysfunction may increase the risk for severe disease as well as hypertension does, but we did not find any difference either; nonetheless, the incidence of pre-eclampsia was higher in our study population in both cases (21.1%) and controls (27.2%) compared to the 6–8% in the general population. It has been reported that COVID-19 can cause manifestations that mimic pre-eclampsia; this fact could explain why pre-eclampsia could have been overdiagnosed. In this matter, it has been previously reported that pre-eclampsia-like syndrome can be adequately differentiated using pro- and anti-angiogenic markers [2,11,25,26,27].

In our study, the mother’s blood group of non-O was an independent risk factor associated with the occurrence of severe–critical COVID-19 during pregnancy (OR 6.29). Although there are few studies reporting this association in pregnancy, it is controversial and seems to depend on the population studied. In this vein, studies conducted in countries where there is great ethnic diversity (white, non-Hispanic black, Latin-American, and Arabic, among others) did not find differences regarding this issue. In contrast, a study that was reported on the risk of severe COVID-19 infection was 3.6 times higher in women with blood group O when they were compared with blood type non-O subjects [21,28,29]. However, our results are consistent with the findings of a recent study, also in Mexican pregnant women, suggesting that the AB blood group in pregnant women is associated with an increased risk of severity and mortality in COVID-19 [15]. In this regard, it has been reported that blood type O is associated with a lower risk of infection because anti-A and or anti-B antibodies are present in individuals with blood type O and could bind to the corresponding antigens on the viral envelope and contribute to viral neutralization [30]. The plausible explanation for this fact is that ACE2 has a similarity between 76 and 78% with the spike (S) protein receptor binding domain of SARS-CoV and SARS-CoV-2, suggesting that they may share a common receptor. ABO antigens could maximize or minimize the binding capacity of the spike protein to host cell surface receptors. Viral entry is facilitated by the interaction of subunit S1 via two domains: S1A, corresponding to the N-terminal region (which interacts with sialic-acid-containing glycoproteins and glycolipids), and S1B, corresponding to the receptor binding domain, which binds to ACE2 receptors. Another mechanism is the lower concentration and biological activity of von Willebrand factor in subjects with type O blood and longer half-life and higher circulating concentrations of von Willebrand factor and factor VIII in people with blood types other than O (A, B, or AB) since it is known that severe COVID-19 infection is associated with a hypercoagulable state [10,31,32].

Finally, we noted that non-vaccination is an important and independent factor associated with disease severity after the start of mass vaccination [13]. Specifically, studies in pregnancy are primarily designed to ensure the safety of the vaccine and to protect both the mother and the newborn from developing serious diseases [17,21]; therefore, several studies recommend vaccination, as it is safe and no serious events have been demonstrated [33,34], which is consistent with our results since in the subgroup of 170 pregnant women vaccinated we found no serious adverse reactions or complications. Nevertheless, while governments should seek and encourage mass vaccination of their populations, they should always respect the principle of autonomy, as we know that there are variables that can influence the acceptance or not acceptance of vaccination, including education, where higher levels of education have been reported to be a strong positive predictor of vaccine acceptance, as well as receiving advice from a gynecologist was reported to be independently associated with SARS-CoV-2 vaccine uptake (OR 2.55) [35,36]. Furthermore, no studies were found that estimated the risk of non-vaccination for severe–critical COVID-19 in pregnancy; our results have shown that this factor is relevant (OR 10.18).

The risk factors identified in our study may be useful for healthcare providers in identifying pregnant women at increased risk of developing severe–critical COVID-19, for advising vaccination of women planning or in early pregnancy, as well as for maintaining preventive strategies and seeking medical attention on time to reduce the transmission and complications of SARS-CoV-2 and other emerging viral pathogens capable of causing epidemics.

The strengths of our study include a large, well-defined population of women infected with SARS-CoV-2 who received the same standardized medical protocol, the recruitment and selection of the population occurred at the diagnostic confirmation (positive RT-PCR) before they were classified as case or control, and the fact that all data were collected from the medical charts which evidently avoided selection and memory bias. Further, to rule out the effects of potential confounders, the estimated ORs were adjusted for established risk factors.

We are aware of the presence of certain limitations, including lack of generalizability to pregnant women at high risk and that some laboratory tests, such as D-dimer and C-reactive protein, were not available for all participants, especially in the control group. Nonetheless, the results reported in this study are representative of the population studied.

## 5. Conclusions

Our results show that non-vaccination, the non-O blood type followed by maternal age > 35 years, chronic hypertension, gestational age at the infection ≥ 31 weeks, and multiparity are independent risk factors associated with the occurrence of severe–critical COVID-19 during pregnancy.

## Figures and Tables

**Table 4:** Risk factors associated with the occurrence of severe–critical COVID-19 during pregnancy.

Variable	Cases Severe–Critical COVID-19 [n = 123]	Controls Non-Severe COVID-19 [n = 635]	Crude OR [CI 95%]	Adjusted OR [CI 95%]
**Mother’s blood type, n [%]**				
O	29 [23.6]	431 [67.9]	Reference	
A	79 [64.2]	182 [28.7]	6.45 [4.07–10.21] *	
B	11 [8.9]	14 [2.2]	11.67 [4.86–28.00] *	
AB	4 [3.3]	9 [1.2]	6.60 [1.91–22.74] *	
**° Pregestational diabetes, n [%]**	10 [8.1]	15 [2.4]	3.64 [1.59–8.30] *	NS
**° Obesity, n [%]**	15 [12.2]	38 [6.0]	2.18 [1.16–4.10] *	NS
**Multiparity, n [%]**	73 [59.3]	206 [32.4]	3.04 [2.04–4.51] *	2.80 [1.71–4.59] *
**Gestational age at infection ≥ 31 weeks, n [%]**	81 [65.9]	197 [31.0]	4.28 [2.84–6.45] *	3.28 [2.01–5.36] *
**Maternal age > 35 years, n [%]**	39 [31.7]	59 [9.2]	4.53 [2.84–7.21] *	5.76 [3.17–10.48] *
**Chronic hypertension, n [%]**	12 [9.8]	13 [2.1]	5.17 [2.3–11.62] *	5.12 [1.83–14.34] *
**Mother’s blood group non-O, n [%]**	94 [76.4]	205 [32.2]	6.79 [4.34–10.64] *	6.29 [3.73–10.60] *
**Non vaccination against COVID-19, n [%]**	115 [95.1]	471 [74.2]	5.00 [2.39–10.47] *	10.18 [4.34–23.86] *

Data are reported as count and frequency, n [%]. * *p*-value < 0.05 was considered statistically significant. Adjusted odds ratios by logistic regression analysis. OR means odds ratio; CI 95%, confidence interval of 95%. ° Pregestational diabetes and obesity had a significant crude OR in the univariate analysis, but not in the multivariate model.

**Table 5:** Risk factors associated with the occurrence of severe–critical COVID-19 during pregnancy in non-vaccinated women.

Variable	Cases Severe–Critical COVID-19 [n = 114]	Controls Non-Severe COVID-19 [n = 472]	Crude OR [CI 95%]	Adjusted OR [CI 95%]
**Mother’s blood group non-O, n [%]**	92 [80.7]	120 [25.4]	12.26 [7.37–20.41] *	9.04 [5.02–16.28] *
**Maternal age > 35 years, n [%]**	37 [32.5]	39 [8.3]	5.33 [3.20–8.89] *	6.08 [3.13–11.78] *
**Chronic hypertension, n [%]**	11 [9.6]	11 [2.3]	4.47 [1.88–10.60] *	5.11 [1.57–16.61] *
**Gestational age at infection ≥ 31 weeks, n [%]**	76 [66.7]	135 [28.6]	4.99 [3.22–7.73] *	3.29 [1.93–5.59] *
**Multiparity, n [%]**	65 [57.0]	108 [22.9]	4.47 [2.91–6.86] *	2.50 [1.47–4.24] *
**° Pregestational diabetes, n [%]**	10 [8.8]	12 [2.5]	3.68 [1.55–8.76] *	NS
**° Obesity, n [%]**	15 [13.2]	26 [5.5]	2.59 [1.32–5.08] *	NS

Data are reported as count and frequency, n [%]. * *p*-value < 0.05 was considered statistically significant. Adjusted odds ratios by logistic regression analysis. OR means odds ratio; CI 95%, confidence interval of 95%. ° Pregestational diabetes and obesity had a significant crude OR in the univariate analysis, but not in the multivariate model.

## Data Availability

Data that support the findings of this study are available from the corresponding author upon reasonable request.

## Abbreviations

COVID-19, coronavirus disease 2019; SARS-CoV-2, severe acute respiratory syndrome coronavirus 2; RT-PCR, reverse transcription polymerase chain reaction; OR, odds ratio; 95% CI, confidence interval at 95%; RASS, renin–angiotensin–aldosterone system; ACE2, angiotensin-converting enzyme 2; Ang 1-7, angiotensin 1-7; Ang II, angiotensin II.

## References

[1] Di Mascio D., Khalil A., Saccone G., Rizzo G., Buca D., Liberati M., Vecchiet J., Nappi L., Scambia G., Berghella V. (2020). Outcome of coronavirus spectrum infections (SARS, MERS, COVID-19) during pregnancy: A systematic review and meta-analysis. Am. J. Obstet. Gynecol. MFM.

[2] Gao Y.D., Ding M., Dong X., Zhang J.J., Kursat Azkur A., Azkur D., Gan H., Sun Y.L., Fu W., Li W. (2021). Risk factors for severe and critically ill COVID-19 patients: A review. Allergy.

[3] Panahi L., Amiri M., Pouy S. (2020). Risks of Novel Coronavirus Disease (COVID-19) in Pregnancy; a Narrative Review. Arch. Acad. Emerg. Med..

[4] Maranto M., Zaami S., Restivo V., Termini D., Gangemi A., Tumminello M., Culmone S., Billone V., Cucinella G., Gullo G. (2023). Symptomatic COVID-19 in Pregnancy: Hospital Cohort Data between May 2020 and April 2021, Risk Factors and Medicolegal Implications. Diagnostics.

[5] Yirmiya K., Yakirevich-Amir N., Preis H., Lotan A., Atzil S., Reuveni I. (2021). Women’s Depressive Symptoms during the COVID-19 Pandemic: The Role of Pregnancy. Int. J. Environ. Res. Public. Health.

[6] McBroom K. (2021). A Comparison of Zika Virus and COVID-19: Clinical Overview and Public Health Messaging. J. Midwifery Womens Health.

[7] Gullo G., Scaglione M., Cucinella G., Riva A., Coldebella D., Cavaliere A.F., Signore F., Buzzaccarini G., Spagnol G., Laganà A.S. (2022). Congenital Zika Syndrome: Genetic Avenues for Diagnosis and Therapy, Possible Management and Long-Term Outcomes. J. Clin. Med..

[8] Gao F., Zheng K.I., Wang X.B., Sun Q.F., Pan K.H., Wang T.Y., Chen Y.P., Targher G., Byrne C.D., George J. (2020). Obesity Is a Risk Factor for Greater COVID-19 Severity. Diabetes Care.

[9] Zhang J.J., Dong X., Liu G.H., Gao Y.D. (2023). Risk and Protective Factors for COVID-19 Morbidity, Severity, and Mortality. Clin. Rev. Allergy Immunol..

[10] Zhang Y., Garner R., Salehi S., La Rocca M., Duncan D. (2021). Association between ABO blood types and coronavirus disease 2019 (COVID-19), genetic associations, and underlying molecular mechanisms: A literature review of 23 studies. Ann Hematol.

[11] WHO (2021). Guidelines Approved by the Guidelines Review Committee. Clinical Management of COVID-19: Living Guideline.

[12] Thompson J.L., Nguyen L.M., Noble K.N., Aronoff D.M. (2020). COVID-19-related disease severity in pregnancy. Am. J. Reprod. Immunol..

[13] World Health Organization (2023). Clinical Management of COVID-19: Living Guideline [Internet].

[14] Allotey J., Stallings E., Bonet M., Yap M., Chatterjee S., Kew T., Debenham L., Llavall A.C., Dixit A., Zhou D. (2020). Clinical manifestations, risk factors, and maternal and perinatal outcomes of coronavirus disease 2019 in pregnancy: Living systematic review and meta-analysis. BMJ.

[15] Sevilla-Montoya R., Helguera-Reppeto A.C., Monroy-Muñoz I.E., Vargas-Pavia T.A., Valdés-Montoya E.I., Solis-Paredes M., Torres-Torres J., Velazquez-Cruz R., Muñoz-Medina J.E., Martinez-Cordero C. (2023). Blood Type Associated with the Risk of COVID-19 Infection in Pregnant Women. Diagnostics.

[16] Samadi P., Alipour Z., Ghaedrahmati M., Ahangari R. (2021). The severity of COVID-19 among pregnant women and the risk of adverse maternal outcomes. Int. J. Gynaecol. Obstet..

[17] Limaye M.A., Roman A.S., Trostle M.E., Venkatesh P., Lantigua Martinez M., Brubaker S.G., Chervenak J., Wei L.S., Sahani P., Grossman T.B. (2022). Predictors of severe and critical disease in pregnant women with SARS-CoV-2. J. Matern. Fetal Neonatal Med..

[18] Zheng C., Shao W., Chen X., Zhang B., Wang G., Zhang W. (2022). Real-world effectiveness of COVID-19 vaccines: A literature review and meta-analysis. Int. J. Infect. Dis..

[19] Kalafat E., Prasad S., Birol P., Tekin A.B., Kunt A., Di Fabrizio C., Alatas C., Celik E., Bagci H., Binder J. (2022). An internally validated prediction model for critical COVID-19 infection and intensive care unit admission in symptomatic pregnant women. Am. J. Obstet. Gynecol..

[20] Jamieson D.J., Rasmussen S.A. (2022). An update on COVID-19 and pregnancy. Am. J. Obstet. Gynecol..

[21] Nana M., Nelson-Piercy C. (2021). COVID-19 in pregnancy. Clin. Med..

[22] Kazemi Aski S., Norooznezhad A.H., Shamshirsaz A.A., Mostafaei S., Aleyasin A., Nabavian S.M., Alimohammadi S., Ahangari R., Ariana S., Ghotbizadeh F. (2022). Clinical features and risk factors associated with acute respiratory distress syndrome in pregnant women diagnosed with COVID-19: A multi-center case-control study. J. Matern. Fetal Neonatal Med..

[23] Kayem G., Lecarpentier E., Deruelle P., Bretelle F., Azria E., Blanc J., Bohec C., Bornes M., Ceccaldi P.F., Chalet Y. (2020). A snapshot of the Covid-19 pandemic among pregnant women in France. J. Gynecol. Obstet. Hum. Reprod..

[24] Lokken E.M., Huebner E.M., Taylor G.G., Hendrickson S., Vanderhoeven J., Kachikis A., Coler B., Walker C.L., Sheng J.S., Al-Haddad B.J.S. (2021). Disease severity, pregnancy outcomes, and maternal deaths among pregnant patients with severe acute respiratory syndrome coronavirus 2 infection in Washington State. Am. J. Obstet. Gynecol..

[25] Mendoza M., Garcia-Ruiz I., Maiz N., Rodo C., Garcia-Manau P., Serrano B., Lopez-Martinez R.M., Balcells J., Fernandez-Hidalgo N., Carreras E. (2020). Pre-eclampsia-like syndrome induced by severe COVID-19: A prospective observational study. BJOG.

[26] Tossetta G., Fantone S., Delli Muti N., Balercia G., Ciavattini A., Giannubilo S.R., Marzioni D. (2022). Preeclampsia and severe acute respiratory syndrome coronavirus 2 infection: A systematic review. J. Hypertens..

[27] Serrano B., Bonacina E., Garcia-Ruiz I., Mendoza M., Garcia-Manau P., Garcia-Aguilar P., Gil J., Armengol-Alsina M., Fernández-Hidalgo N., Sulleiro E. (2023). Confirmation of preeclampsia-like syndrome induced by severe COVID-19: An observational study. Am. J. Obstet. Gynecol. MFM.

[28] Sainz Bueno J.A., Cerrillos González L., Abascal-Saiz A., Rodríguez Gallego M.V., López Pérez R., Fernández Alonso A.M., de la Cruz Conty M.L., Alonso Saiz R., Molina Oller M., Santamaría Ortiz A. (2021). Association of ABO and Rh blood groups with obstetric outcomes in SARS-CoV-2 infected pregnancies: A prospective study with a multivariate analysis. Eur. J. Obstet. Gynecol. Reprod. Biol..

[29] Arslan B., Bicer I.G., Sahin T., Vay M., Dilek O., Destegul E. (2022). Clinical characteristics and hematological parameters associated with disease severity in COVID-19 positive pregnant women undergoing cesarean section: A single-center experience. J. Obstet. Gynaecol. Res..

[30] Goel R., Bloch E.M., Pirenne F., Al-Riyami A.Z., Crowe E., Dau L., Land K., Townsend M., Jecko T., Rahimi-Levene N. (2021). ABO blood group and COVID-19: A review on behalf of the ISBT COVID-19 Working Group. Vox Sang..

[31] Hoiland R.L., Fergusson N.A., Mitra A.R., Griesdale D.E.G., Devine D.V., Stukas S., Cooper J., Thiara S., Foster D., Chen L.Y.C. (2020). The association of ABO blood group with indices of disease severity and multiorgan dysfunction in COVID-19. Blood Adv..

[32] Liu N., Zhang T., Ma L., Zhang H., Wang H., Wei W., Pei H., Li H. (2021). The impact of ABO blood group on COVID-19 infection risk and mortality: A systematic review and meta-analysis. Blood Rev..

[33] Prabhu M., Riley L.E. (2023). Coronavirus Disease 2019 (COVID-19) Vaccination in Pregnancy. Obstet Gynecol.

[34] Zdanowski W., Markiewicz A., Zdanowska N., Lipińska J., Waśniewski T. (2022). Tolerability of the BNT162b2 COVID-19 Vaccine during Pregnancy among Polish Healthcare Professionals. Vaccines.

[35] Maranto M., Gullo G., Bruno A., Minutolo G., Cucinella G., Maiorana A., Casuccio A., Restivo V. (2023). Factors Associated with Anti-SARS-CoV-2 Vaccine Acceptance among Pregnant Women: Data from Outpatient Women Experiencing High-Risk Pregnancy. Vaccines.

[36] Montanari Vergallo G., Del Rio A., Negro F., Zaami S. (2022). COVID-19 vaccine mandates: What are the current European public perspectives?. Eur. Rev. Med. Pharmacol. Sci..

